# Intraperitoneal chemotherapy of the peritoneal surface using high-intensity ultrasound (HIUS): investigation of technical feasibility, safety and possible limitations

**DOI:** 10.7150/jca.48519

**Published:** 2020-10-18

**Authors:** Hien Lau, Tanja Khosrawipour, Agata Mikolajczyk, Piotr Frelkiewicz, Jakub Nicpon, Mohamed Arafkas, Alessio Pigazzi, Wolfram Trudo Knoefel, Veria Khosrawipour

**Affiliations:** 1 Division of Colorectal Surgery, Department of Surgery, University of California Irvine, Orange, USA; 2 Department of Surgery (A), University-Hospital Düsseldorf, Heinrich-Heine University Düsseldorf, Germany; 3 Department of Biochemistry and Molecular Biology, Faculty of Veterinary Medicine, Wroclaw University of Environmental and Life Sciences, Wroclaw, Poland; 4 The Center of Experimental Diagnostics and Innovative Biomedical Technology, Wroclaw University of Environmental and Life Sciences, Wroclaw, Poland; 5 Department of Plastic Surgery, Ortho-Clinic Dortmund, Dortmund, Germany

**Keywords:** Intraperitoneal chemotherapy, high-intensity ultrasound, HIUS, peritoneal, surface

## Abstract

**Introduction:** The penetration of chemotherapeutic drugs into peritoneal nodules remains at levels well below 1 mm, thus significantly limiting the antitumor effect of intraperitoneal chemotherapy (IPC). Recently, high-Intensity ultrasound (HIUS) has been discovered as a potential tool to significantly improve peritoneal diffusion rates. Despite promising preliminary data, basic aspects regarding its technical feasibility, safety and possible limitations remain unclear. This study aims to enhance our current understanding of HIUS and test its applicability using an ex-vivo swine model.

**Methods:** Three postmortem swine were subject to laparotomy and consecutive lavage with 0.9%NaCl saline and HIUS application. For this purpose, a large HIUS radiating pen was introduced into the abdominal cavity and HIUS was applied on two of the four abdominal quadrants for 300 seconds each at an output power of 70 W, 50 % amplitude and 20 kHz frequency. Following the procedure, small intestinal tissue samples were retrieved for further analyses.

**Results:** Peritoneal and subperitoneal layers showed structural changes only visible on a microscopic level. The peritoneal layer was transformed into a mesh-like structure while the subperitoneal layer (depth of 142 +/- 28 µm) exhibited microcavities and vascular detachment from surrounding tissues. No bowel rupture or vascular perforations were observed.

**Conclusions:** Our data indicate that HIUS is a technically feasible and safe add-on procedure for intraperitoneal chemotherapy (IPC) with measurable microscopic changes on the peritoneal surface. Pretreatment of the abdominal cavity with HIUS could significantly improve IPC efficacy. Further studies are required to optimize and evaluate this novel approach.

## Introduction

Peritoneal metastases (PM) is a common manifestation of advanced gastrointestinal and gynecological cancers. Since the penetration of chemotherapeutic drugs into peritoneal tumor nodules is well below < 1 mm, this significantly limits the antitumor effect of intraperitoneal chemotherapy (IPC) 1, 2. Various attempts have been made to improve drug availability within these tumor nodules. Hyperthermia 3 and intraperitoneal pressure 4 are two physical concepts used in IPC which have, to some degree, improved drug penetration into tumor tissue. Hyperthermic intraperitoneal chemotherapy (HIPEC) combined with cytoreductive surgery 5 is an example of a PM treatment based the concept of hyperthermia. On the other hand, pressurized intraperitoneal aerosol chemotherapy (PIPAC) is a therapy targeting more advanced PM based on the concept of pressure 6, 7. Clinical and experimental studies have also tested whether irradiation 8 - 10 and new drug formulas 11 - 13 may potentially increase chemotherapeutic drug penetration.

While many attempts have been made to improve penetration rates, and most approaches demonstrated limited efficiency, some of these new concepts were able to establish themselves in the clinical setting. Despite these efforts, penetration levels remain at below < 500 µm 15 - 17. Thus, there is a pressing need to develop methods with improved drug delivery resulting in increased drug penetration depths. Preliminary data on high-intensity ultrasound (HIUS) are promising and indicate its potential to increase in-depth tissue penetration of applied substances 18. While the addition of HIUS results in a threefold increased drug penetration compared to conventional IPC without HIUS, the feasibility of its application within the abdominal cavity remains unclear.

In previous ex-vivo models, HIUS has been applied to the parietal peritoneum either by a transcutaneous approach or at some distance from the tissue. Thus, until today, HIUS has not been tested in an abdominal model which would be an important step to assess this application's safety. By means of this study, we aim to explore possible side effects of HIUS including physical damage to tissues and internal organs within the abdominal cavity. Since the current technology offers HIUS mostly in the form of pen devices emitting ultrasonic radiation waves, it is crucial to assess possible risks of intestinal rupture by direct contact with such devices. With over two decades of application in solid tumor therapy with successful results in some cases 19 - 21, HIUS is a promising tool which provides unique advantages including low invasiveness and absence of radiation. The aim of this study is to evaluate whether it is safe to use HIUS as a supplementary tool to prepare the abdominal cavity for IPC. Moreover, this study aims to investigate HIUS' effects on the small intestine and evaluate the risk for intestinal ruptures or vascular damage following HIUS application.

## Material and Methods

### Postmortem study

**Figure 1:**
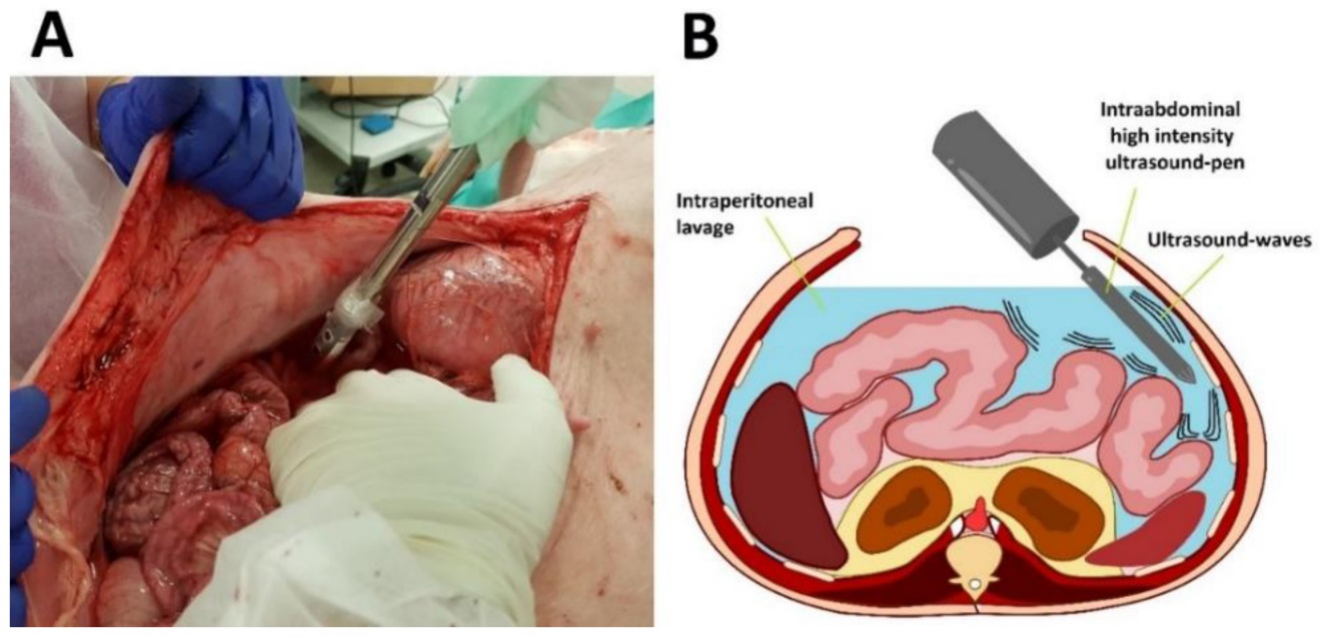
Application of high-intensity ultrasound (HIUS) using a large pen during laparotomy. Left: Laparotomy on swine with saline filled abdominal cavity. Right: Illustration of the procedure

Experiments were performed on three swine (commercially obtained from local pork supplier, Zerniki Wielkie/ Polish large white breed pigs) at 30 minutes postmortem. Swine were premedicated with an intramuscular injection of midazolam (0.1 mg/kg, Midanium 5 mg/ml, WZF Polfa S.A., Poland), medetomidine (0.02 mg/kg, Cepetor 1 mg/ml, CP-Pharma Handelsgesellschaft, Germany) and ketamine mixture (8 mg/kg, Ketamina 100 mg/ml, Biowet Puławy sp. z o.o., Poland). Following the experiments, swine were euthanized with an intravenous injection using sodium pentobarbital with pentobarbital (50mg/kg with 12 mg/kg, Morbital 133.3 mg/ml + 26.7 mg/ml, Biowet Pulawy Sp. z o.o., Poland). Cadavers were placed in a supine position. Next, after a median laparotomy was performed across the midline, 4 liters of conventional saline solution were introduced into the abdominal cavity. The abdomen was formally divided into 4 quadrants. 2 quadrants received HIUS while the other 2 quadrants were used as control. A large HIUS pen was manually placed into the abdominal cavity and ultrasound was applied with a metal pen using a sonicator (Bandelin Sonoplus, UW 2070) (Figure 1 A and B). The tip of the pen was held into the abdominal lavage. A considerably large dose of HIUS was applied to each quadrant for 300 seconds, respectively. Each treatment consisted of 0.3 seconds of active and 0.7 seconds of passive intervals, with 20 kHz frequency, output power of 70 W and 50 % of amplitude. After the procedure, tissue samples from each quadrant were retrieved and further analyzed.

### Microscopic analysis

After the procedure, samples of the small intestine were removed from all quadrants and embedded in paraffin prior to standard hematoxylin and eosin (H&E) staining. Analyses were performed using light microscopy (Olympus life Sciences U-HGLGPS).

### Statistical analyses

Experiments were independently performed three times. A total of 12 tissue sections per tissue sample were subjected to doxorubicin penetration measurements. Prism 7.0 software (GraphPad, La Jolla, CA, USA) was used to analyze the data. A student-t test was used for group analyses. A significant p-value was considered at p <0.05.

### Ethical statement

Surgical procedures on the swine were performed at the Center for Experimental Diagnostics and Innovative Biomedical Technology, Wroclaw, Poland. Experiments were performed in the morning at 10 a.m. Approval of the local board on animal welfare was obtained (Zapytanie 8/8/2019) according to the Polish law. The study included three, approximately 40-kilogram, female, 60-day-old swine of Polish large white breed pig (domestic pig by local pork supplier, Zerniki Wielkie). Swine were housed in a concrete stable of the following dimensions: 1.8m of width and 2.5m of length. Swine were housed at room temperature of 18-20°C and relative humidity of 60-75% maintained by air condition. Stables were cleaned twice a day, swine were fed a balanced diet and had unlimited access to water. Food was restricted for 12h and water restricted for 4h before anesthesia. Soft balls, rope and wood logs as well as music were provided for environmental enrichment. All swine received humane care in compliance with the 8^th^ edition of the Guide for the Care and Use of Laboratory Animals published by the National Institutes of Health 22.

## Results

### Peritoneal changes

**Figure 2:**
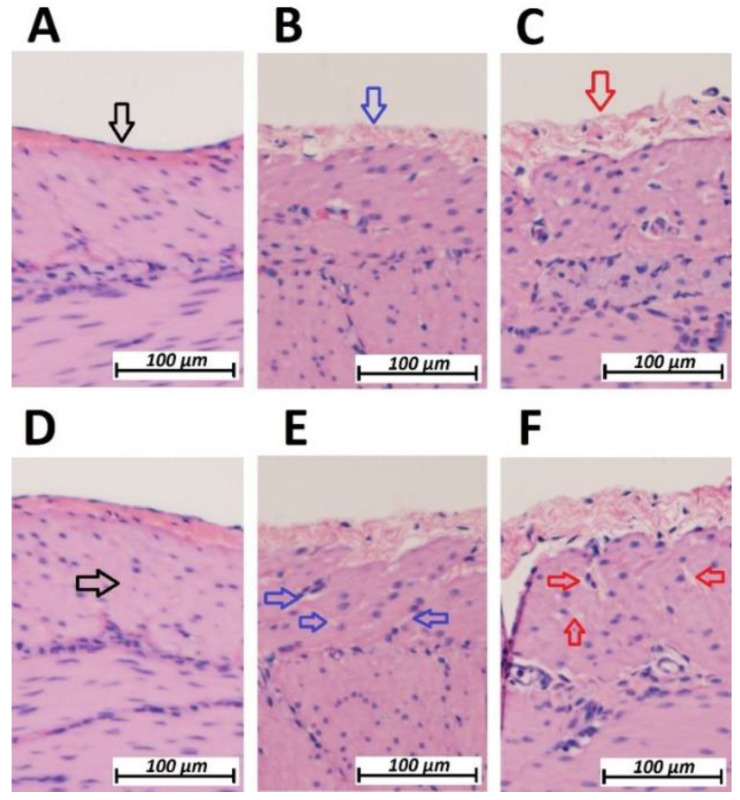
Microscopic analysis of the peritoneal structure of swine small intestine after HIUS. (Hematoxylin and Eosin (H&E) staining). A: Control (No HIUS). B: low grade changes with HIUS. C: high grade changes with HIUS. Upper pictures: increasing transformation of peritoneal layer from normal (black arrow) to “mesh-like” layer (red arrow). Lower pictures: increasing microcavitation of the subperitoneal layer from normal compact tissue (black arrow) to a cavity-rich tissue (red arrow)

**Figure 3:**
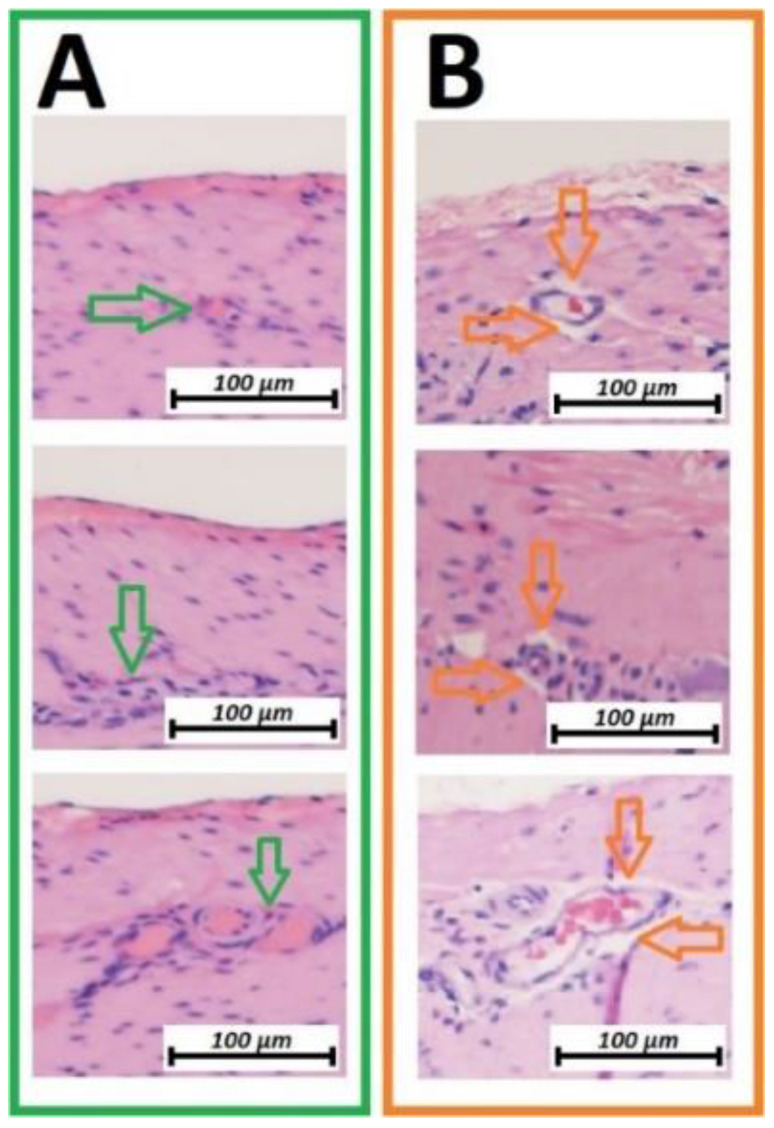
Microscopic analysis of the vascular structure of swine small intestine after HIUS (Hematoxylin and Eosin (H&E) staining). Isolation of vascular system from the surrounding tissue. Vascular structures remain microscopically intact. A (green): Control, no HIUS, B: (orange) changes following HIUS

**Figure 4:**
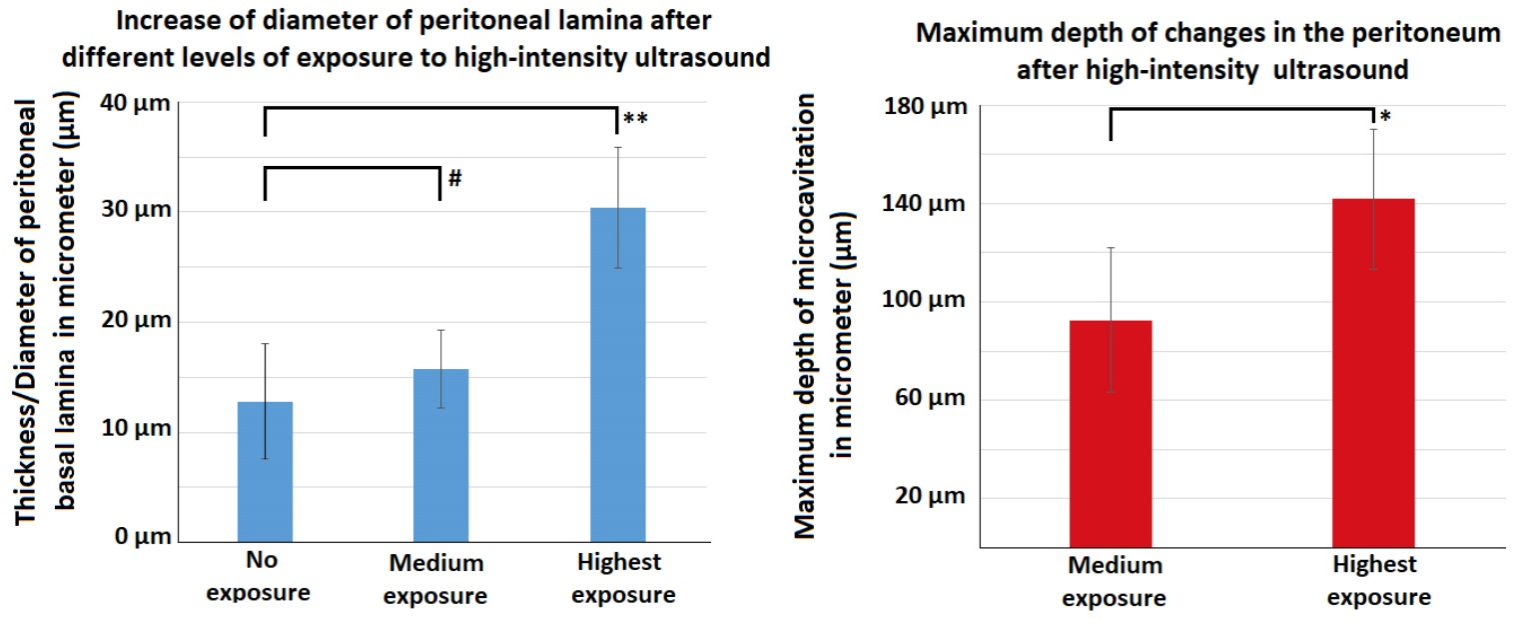
Effects of HIUS on the small intestine in a laparotomy model. Left: Increase of peritoneal lamina diameter at different exposure levels. Right: Maximum depth of observed tissue changes following HIUS

Following HIUS application, no bowel rupture or other macroscopic disruptions of the intestinal organs were observed. Also, no residual bleeding or other damages to the vascular intestinal structures were detected. The texture of the intestinal surface did not macroscopically change as previously reported in other models 21. Small bowel tissue samples showed significant microscopic structural changes. The peritoneal endothelium itself was transformed from a compact tissue layer to a homogenous mesh structure (figure 2). As a result, the peritoneal layer was almost twice the volume of the untreated control. The diameter of the peritoneal endothelium increased from approximately 13 +/- 5 µm to 16 +/- 4 µm up to 30 +/- 6 µm in samples with maximum HIUS exposure. However, the structural integrity of the peritoneum remained intact and no “bite mark” lesions were observed in the peritoneal layer. This transformation into mesh-like structures was possibly proportional to the intensity of the applied HIUS, with varying degrees of peritoneal “swelling” within samples (figures 2 and 4). The underlying subperitoneal layer was filled with microcavities of increasing size (figures 2 and 3) which are consistent with observed effects on the overlying peritoneal endothelium. The appearance of these microcavities under the peritoneal layer were limited to the first 92 +/- 30 µm and 142 +/- 28 µm after 1 minute and 3 minutes, respectively. While the underlying longitudinal muscular structures were affected, circular muscles tissue were not.

### Vascular changes

Microcavitation was more prominent on the vascular structures between linear and circular muscle tissues. Vascular structures were practically detached from the surrounding surface (figure 3B). These microcavitations around the vessels were not associated with any visible or detectable disruptions of the vascular structures themselves. With changes strictly limited to the first vascular network located between muscle groups, no changes were detectable on the deeper vascular network on the luminal side of the small intestine.

## Discussion

Despite advances in chemotherapeutic regimens and new drug compositions, poor response to both systemic and local treatment is observed in many patients. This is mainly attributed to molecular mechanisms and limited drug distribution in tumor tissues 1, 23. However, attempts to improve the antitumoral response were only partially successful in new IPC approaches 24 - 26. Changes in PIPAC treatment parameters for example only modestly improved penetration rates 4,14, while the addition of irradiation or applicational modifications 10 did not at all improve performance. However, we know that increasing tissue penetration enhances the antitumor effect with a higher local drug disposition 1. In previous studies, HIUS has demonstrated substantially enhanced drug penetration in the peritoneal tissue 18. In the clinical setting, HIUS is increasingly used as a non-invasive treatment for both primary and metastatic tumors.

Besides its previously described effects, HIUS has additional antitumor effects including ablation and mechanical disruption of tumor tissues 27, 28. HIUS is a versatile tool used in the treatment of uterine fibroids 29, various solid tumors of pancreas, liver, renal system and prostate, and breast cancer 30 - 33. However, the physical energy transported by HIUS might cause severe side effects. In our study, the applied doses resulted in only limited impact on the structural integrity of the small intestine. Following our observations, it appears as if a large amount of energy is absorbed on the peritoneal surface without altering the structural integrity of the deeper tissues. Moreover, structural effects do not seem to disrupt the cell plasma in most cells. This is an especially important finding since extensive cell disruption would otherwise lead to local necrosis. The intracellular cytoskeleton might play a role in stabilizing the cell membrane, an astonishing finding considering that liposomal particles cannot withstand similar doses of HIUS 21. The disruptive effects of HIUS seem to primarily focus on the extracellular fibers in the peritoneal tissue as well as on the muscle fiber construction of the underlying tissue.

However, as our study indicates, this effect remains on a microscopical level and is thus limited in depth. Previous HIUS studies were always restricted to the analysis of solid parietal peritoneal samples in a small box model. While they offered important preliminary data on HIUS application, it remained unclear whether HIUS could cause bowel perforation or vascular ruptures. Additionally, there were uncertainties regarding the technical use of HIUS in an anatomic model with sensitive intraabdominal structures. Intraabdominal HIUS application requires a new applicational device optimized for both laparoscopic and laparotomy approaches.

In addition to effects on the peritoneal layer, it is interesting that HIUS seems to detach the vascular system from the surrounding tissue. This effect had not been observed before. Previous research on tissue permeabilization mechanisms during HIUS attribute this effect to the creation of micro gas bubbles 34,35. While this finding could also explain microcavitation effects around vascular structures, it can also be of great importance for IPC and intravenous chemotherapeutic treatments. In fact, HIUS demonstrated to increase blood circulation in targeted tissues and improve perfusion of chemotherapeutic agents in liver tumors and glioblastoma 36, 37. This effect can be especially important in patients with peritoneal cancer if HIUS is combined with systemic chemotherapy. While our model unfortunately does not cover this aspect, this requires further study and individual investigation. Additionally, the current HIUS pen requires an update to ensure easier handling during intraabdominal use. However, we believe that HIUS in combination with different forms of chemotherapy has the potential for intraabdominal use and can be a practical option to improve current treatment regimens. However, its effects on the abdominal cavity and the peritoneum need to be studied more closely by means of in-vivo models.

## Conclusion

Our data indicate that intraperitoneal HIUS can be a safe and feasible tool in overcoming current limitations in IPC in combination with either HIPEC, PIPAC or any other form of IPC. HIUS prepares the peritoneum for the penetration of chemotherapeutic substances but does not cause imminent macroscopic tissue disruption which could rupture the intestinal wall. The effects on cellular structures seem limited as extracellular and collagenous matrix are more susceptible to changes following HIUS. Further studies are required to validate these preliminary findings and develop HIUS for possible clinical application in PM via IPC.

## Figures and Tables

**Figure 5:**
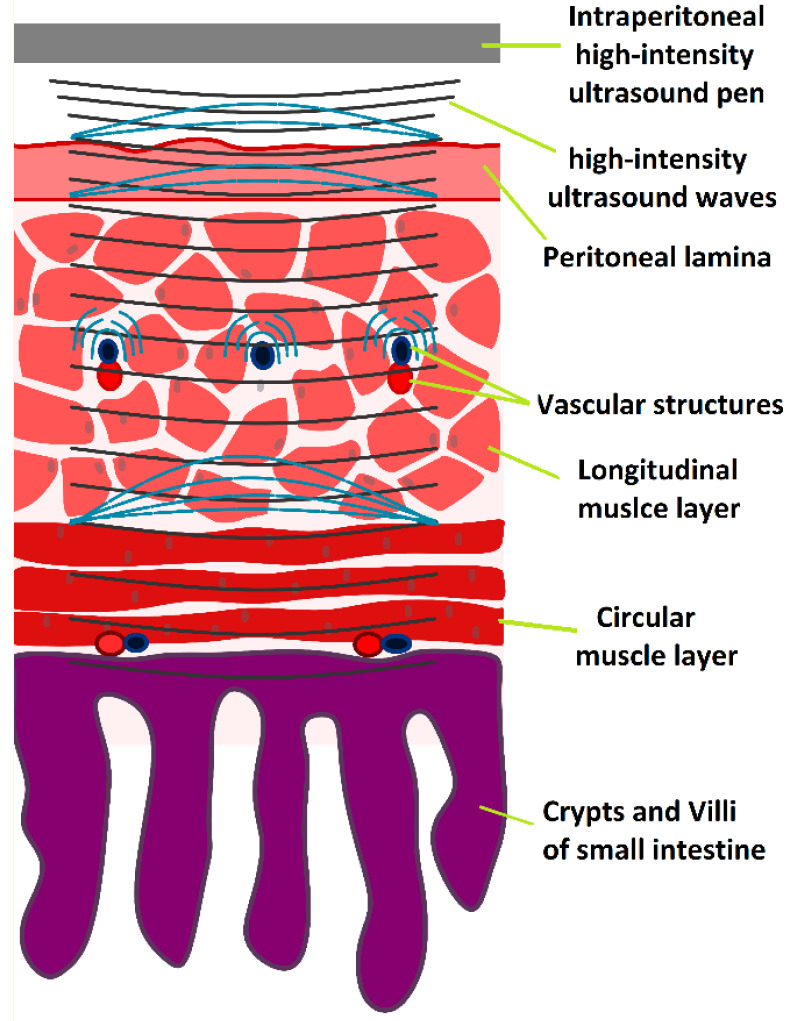
HIUS model penetrating the small intestine with suspected reflection points and areas of high structural stress
